# Effects of phosphorus deficiency on the absorption of mineral nutrients, photosynthetic system performance and antioxidant metabolism in *Citrus grandis*

**DOI:** 10.1371/journal.pone.0246944

**Published:** 2021-02-17

**Authors:** Xin Meng, Wei-Wei Chen, Yan-Yu Wang, Zeng-Rong Huang, Xin Ye, Li-Song Chen, Lin-Tong Yang

**Affiliations:** College of Resources and Environment, Fujian Agriculture and Forestry University, Fuzhou, China; University of Hyderabad School of Life Sciences, INDIA

## Abstract

Phosphorus (P) is an essential macronutrient for plant growth, development and production. However, little is known about the effects of P deficiency on nutrient absorption, photosynthetic apparatus performance and antioxidant metabolism in citrus. Seedlings of ‘sour pummelo’ (*Citrus grandis*) were irrigated with a nutrient solution containing 0.2 mM (Control) or 0 mM (P deficiency) KH_2_PO_4_ until saturated every other day for 16 weeks. P deficiency significantly decreased the dry weight (DW) of leaves and stems, and increased the root/shoot ratio in *C*. *grandis* but did not affect the DW of roots. The decreased DW of leaves and stems might be induced by the decreased chlorophyll (Chl) contents and CO_2_ assimilation in P deficient seedlings. P deficiency heterogeneously affected the nutrient contents of leaves, stems and roots. The analysis of Chl *a* fluorescence transients showed that P deficiency impaired electron transport from the donor side of photosystem II (PSII) to the end acceptor side of PSI, which showed a greater impact on the performance of the donor side of PSII than that of the acceptor side of PSII and photosystem I (PSI). P deficiency increased the contents of ascorbate (ASC), H_2_O_2_ and malondialdehyde (MDA) as well as the activities of superoxide dismutase (SOD), catalase (CAT), ascorbate peroxidase (APX), dehydroascorbate reductase (DHAR) and glutathione reductase (GR) in leaves. In contrast, P deficiency increased the ASC content, reduced the glutathione (GSH) content and the activities of SOD, CAT, APX and monodehydroascorbate reductase (MDHAR), but did not increase H_2_O_2_ production, anthocyanins and MDA content in roots. Taking these results together, we conclude that P deficiency affects nutrient absorption and lowers photosynthetic performance, leading to ROS production, which might be a crucial cause of the inhibited growth of *C*. *grandis*.

## Introduction

Phosphorus (P) is an essential macronutrient for the normal growth of plants. It is not only a key component of macromolecules, such as proteins, nucleic acids, the plasma membrane, ATP, vitamins and some secondary compounds but also plays critical roles in the metabolism of nitrogen compounds, carbohydrate transportation, carbohydrate metabolism and fat metabolism [1]. It also plays an important role in signal transduction and photosynthesis in plants and has a decisive impact on stress resistance, plant dependency on vesicular-arbuscular mycohrrhizal for P uptake in order to produce its maximum growth, yield and quality of crop [2]. In tropical and subtropical areas, P deficiency has become one of the main limiting factors for crop growth due to the loss of P nutrient caused by high temperature and heavy rain, and the fixation of P by iron and aluminum oxides in the soil [1]. Approximately 30% − 40% of the world’s cultivated land is lack of P [3]. Furthermore, inorganic P readily forms complexes with oxides and hydroxides of iron and aluminum in acidic soils and with calcium in alkaline soils, with up to 80% of P applied as fertilizer becoming unavailable for most crops [4]. Such situation is aggravated by inadequate and imbalanced use of fertilizers, leading to reduced nutrient availability for crop growth. P is often a limiting factor because most of P in soil exists as unavailable form for plants. As a result, P fertilizer is heavily applied in agriculture, causing a series of problems, such as heavily increased production cost, rapid depletion of nonrenewable P resource and waterway eutrophication. In addition, inorganic phosphate rocks, which are nonrenewable resources, will be exhausted within the next few centuries [5]. In response to P limitation, plants have evolved various biochemical, metabolic, and morphological adaptations to enhance P acquisition, including increased synthesis and secretion of organic acids and acid phosphatases (APs); higher root/shoot ratio and lateral root growth; and increased root hair length and density for a larger root surface area [1].

As the available literatures indicated, P deficiency significantly decreased the DW of roots and shoots of lettuce and tomato, and the leaf number of Chinese milk vetch, alfalfa, lettuce, tomato and marigold [6]. P deficiency caused a significant reduction in net photosynthesis rate and energy capture efficiency of the photosystem II (PSII) reaction center in rice, sunflower and maize [7–9]. The P-deficiency-induced reduction of photosynthesis rate was also reported in sugar beet [10], soybean [11], tobacco [12], *Zizania latifolia* [13], oat [5], sheepgrass [14], barley [15] and tea [16]. As result, the grain yield or production of crops was compromised by P deficiency [9,17,18]. Short-term P deprivation not only reduced P concentration but also decreased the total chlorophyll (Chl) and carotenoid content in tomato seedlings [19].

P deficiency can alter the metabolism and translocation of carbohydrates, such as soluble sugars and organic acids [5,20–22]. Increased accumulation of carbohydrates, especially sucrose, was observed in leaves of many plant species under P deficiency [5,23–26], which was attributed to low sink demand and limited leaf expansion under P starvation [12,27]. Secretion of organic acids is one of the most important low-P responses in plants, which dissolves soil P via acidification and complexation, and confers differing levels of low-P tolerance in crops [26,28]. Induced organic acid exudation has been found in many plants, including tea [16], white lupin [20], soybean [22], barley [26], and Chinese fir [28], under low-P conditions. Further evidence has revealed that the internal organic acid metabolism may also be involved in the low-P response, as citric, malic and succinic acid concentrations were significantly higher in low-P alfalfa roots [29].

Similar to other abiotic stresses, P deficiency inevitably causes increased production of reactive oxygen species (ROS) as byproducts of photosynthesis. A feedback inhibition mechanism via sugar accumulation leads to lower utilization of the photosynthetic electron transport chain. ROS, including singlet oxygen (^1^O_2_), superoxide anion (O_2_^·−^), hydrogen peroxide (H_2_O_2_) and hydroxyl radical (HO^−^), are partially reduced or excited forms of atmospheric oxygen and can be produced in organelles of aerobic respiration [30]. As a strategic response, plants have evolved highly regulated enzymatic and nonenzymatic mechanisms, which include antioxidant enzymes such as superoxide dismutase (SOD), peroxidase (POD) and catalase (CAT), and antioxidants such as ascorbate (ASC) and reduced glutathione (GSH), to scavenge ROS, keeping a balance between ROS production and elimination [30,31]. Our previous work showed that imbalance between light capture and utilization typically triggers the production of ROS in *C*. *grandis* and/or *C*. *sinensis* under Mg deficiency, B deficiency and Al stress [30,32,33]. P deficiency-induced increases in ROS bursts and scavenging antioxidant systems have been reported in maize [34], rice [8,35], tomato [19], sheepgrass [14], *Arabidopsis* [36], *Phaseolus vulgaris* [37] and tea [38].

*Citrus* is a evergreen fruit tree cultivated in tropical and subtropical areas where the soil is acidic or slightly acidic. According to our previous research based on 319 citrus orchards in southern China, soil acidification has become one of the constraining factors that limited the availability of P to citrus growth and production, especially in newly built orchards [39]. So far, the researches about the effects of P deficiency on plants were more focused on legume and Gramineae crops, but less on woody plants. Investigating the effects of P deficiency on the physiological performance, including nutrient absorption, photosynthesis efficiency and antioxidant metabolism of citrus seedlings, can help us further understand how citrus plants adapt to low P conditions.

## Materials and methods

### Plant culture and P deficiency treatments

Uniform seeds of ‘sour pummelo’ (*Citrus grandis*) were collected from Fujian Academy of Forestry Sciences, Fuzhou, China and were sown in plastic trays containing clean river sand and kept moist by spraying tap water. The seeds were cultured in a green house under natural temperature and light at Fujian Agriculture and Forestry University. After sprouting, the seedlings of ‘sour pummelo’ were irrigated with nutrient solution every other day. Eight weeks later, uniform seedlings with one sprout and two leaves were transplanted to 6-L pottery pots (two seedlings per pot) containing clean river sand and irrigated with nutrient solution until saturated every other day. There were 20 pots for each treatment (two seedlings per pot) in a completely randomized design. The nutrient solution contained the following macronutrients (in mM): KNO_3_, 2.5; Ca(NO_3_)_2_, 2.5; KH_2_PO_4_, 0.2; MgSO_4_, 1; and micronutrients (in μM): H_3_BO_3_, 10; MnCl_2_, 2; ZnSO_4_, 2; CuSO_4_, 0.5; (NH_4_)_6_ Mo_7_O_24_, 0.065; and FeSO_4_-EDTA, 20. Seven weeks after transplantation, each pot was supplied with nutrient solution with or without 0.2 mM KH_2_PO_4_ until saturated every other day for 16 weeks. The pH of the nutrient solution was adjusted to 4.5 with HCl. At the end of the experiment, fully expanded leaves (approximately ten weeks old) from different replicates and treatments were collected and used for all measurements. Root apices (approximately 5 mm) were excised and collected from seedlings. All the samples were wrapped in aluminum foil, frozen in liquid nitrogen and stored at −80 °C until extraction and analysis.

### Plant DW and nutrient measurements

Eight plants per treatment (8 replicates) from different pots were harvested and sliced into shoots and roots. The harvested tissues (all the leaves, stems and roots) were fixed at 120 °C for 20 min and dried at 70 °C for 48 h, and the DWs were measured. Dried roots and leaves were ground into fine powder with a pulverizer and sifted through a 0.15 mm sieve. To measure the root and leaf P, potassium (K), iron (Fe), magnesium (Mn), copper (Cu), zinc (Zn), calcium (Ca), and magnesium (Mg) contents, approximately 0.2 g samples were weighted using electronic balance and digested in a 6 mL mixture of HNO_3_:H_2_O_2_ (5:2 v/v). P was determined using the ammonium molybdate/ascorbic acid spectrophotometric assay [40]. K was assayed using FP640 Flame Photometry (Shanghai Precision Scientific Instrument Co., Ltd, Shanghai, China). Fe, Mn, Cu, Zn, Ca, and Mg were determined using a PinAAcle 900F Atomic Absorption Spectrometer (Perkinelmer Singapore Pte Ltd, Singapore). N was measured using a Kjeltec 8200 Auto Distillation unit (FOSS Analytical AB, Höganäs, Sweden) after samples were digested with H_2_SO_4_ and H_2_O_2_ [41]. Boron (B) was determined by the curcumin method after samples were ashed at 500°C for 5 h and dissolved in 0.1 M HCl [42]. Sulfur (S) was measured using the simple turbidimetric method based on the formation of BaSO_4_ precipitate in colloid form [41]. There were three replicates for element measurement.

### CO_2_ assimilation, Chl *a* fluorescence and Chl content measurements

Gas exchange parameters were determined using a CIRAS-2 portable photosynthesis system (PP Systems, Herts, U.K.) with a PLC6 (U) automatic universal leaf cuvette at an artificial CO_2_ concentration (380 μmol mol^–1^) supplied by a CO_2_ gas cylinder. The photosynthetic photon flux (PPF) was maintained at a constant intensity of 1000 μmol m^–2^ s^–1^ by LED light. The experiment was carried out between 10:00 am and 12:30 pm on a clear day. During measurement, leaf temperature and calculated ambient relative humidity were 28 ± 0.8 °C and 62 ± 1.5%, respectively. Chl *a* fluorescence of dark-adapted leaves was measured using a Handy PEA fluorometer (PP Systems, Herts, U.K.). The parameters, formulae and their description using data extracted rom the Chl *a* fluorescence (OJIP) transient are listed in S1 Table. Leaf Chl *a* and Chl *b* were measured according to the method described by Lichtenthaler [43] after they have been extracted with 80% (v/v) acetone in darkness. There were five replicates for CO_2_ assimilation, six replicates for Chl *a* fluorescence and three replicates for Chl *a* and Chl *b* measurement, respectively.

### Analysis of malondialdehyde (MDA), H_2_O_2_, anthocyanins and antioxidant metabolite levels and antioxidant enzyme activity

MDA was determined according to the method described by Hodges et al. [44] and represented as TBA reactive substances (TBARS). H_2_O_2_ production was assayed according to the method described by Chen et al. [45]. Briefly, eight to ten root apices were incubated in 1.5 mL 50 mM K_2_HPO_4_-KH_2_PO_4_ buffer solution (pH 7.0) containing 0.05% (w/v) guaiacol and 2 U horseradish peroxidase for 2 h at room temperature. Absorbance was measured at 470 nm with a spectrophotometer. The content of H_2_O_2_ in roots and leaves was calculated with an extinction coefficient of ε = 26.6 cm^−1^ mM^−1^. Anthocyanins were measured by the method described by Lee [46].

Guaiacol peroxidase (GuPX), superoxide dismutase (SOD), ascorbate peroxidase (APX), monodehydroascorbate reductase (MDHAR), dehydroascorbate reductase (DHAR), glutathione reductase (GR), glutathione peroxidase (GlPX) and catalase (CAT) were extracted with 50 mM KH_2_PO_4_-KOH solution (pH 7.5) containing 0.3% (w/v) Triton X-100, 0.1 mM EDTA-Na_2_ and 4% (w/v) insoluble polyvinylpolypyrrolidone (PVPP). SOD activity was assayed according to the method described by Giannopolitis and Rice [47]. The reaction mixture contained 1.3 μM riboflavin, 13 mM methionine, 63 μM NBT, 0.05 M Na_2_CO_3_, and appropriate volume of extract. GuPX, APX, CAT, MDHAR, DHAR and GR activities were measured according to the methods described by Chen et al. [48]. GuPX was assayed in 1.0 mL reaction mixture containing 100 mM potassium phosphate buffer (pH 6.0), 16 mM guaiacol, 5 μL 10% (v/v) H_2_O_2_, and the enzyme extract. APX was assayed in 1.0 mL reaction mixture containing 50 mM Hepes-KOH (pH 7.6), 0.1 mM EDTA, 0.2 mM H_2_O_2_, 0.5 mM AsA and appropriate enzyme extract. CAT was assayed in 1.0 mL reaction mixture containing 100 mM potassium phosphate buffer (pH 6.0), 10 μL 10% (v/v) H_2_O_2_ and the enzyme extract. GlPX was measured according to the methods described by Fujita and Hossain [49] and Hasanuzzaman et al. [50].

Reduced glutathione (GSH) and oxidized glutathione (GSSG) were extracted with ice-cold 5% (w/v) trichloroacetic acid (TCA) and measured according to the method described by Griffith [51]. Reduced ascorbate (ASC) and dehydroascorbate (DHA) were extracted with ice-cold 6% (v/v) HClO_4_ and measured according to the method described by Chen et al. [48]. There were four replicates for each measurement of MDA and H_2_O_2_ production, antioxidant metabolites and antioxidant enzymes.

### Quantitative RT-PCR (qRT-PCR) analysis of genes related to the absorption and allocation of K, B and S in *C*. *grandis* roots

The gene special primer pairs of high-affinity potassium transporter protein 1 (*HKT1*), *HKT2*, potassium channel SKOR (*SKOR*, outward rectifying channel), potassium channel AKT1 (*AKT1*), nodulin-like intrinsic protein (*NIP5*.*1*), boron transporter 1 (*BOR1*, outward rectifying channel), high affinity sulfate transporter 1.3 (*HSAT1*.*3*, outward rectifying channel), and high affinity sulfate transporter 1.1 (*HSAT1*.*1*) were designed using Primer Premier (Version 5.0) according to the corresponding sequences deposited in the HZAU citrus genome database (http://citrus.hzau.edu.cn/orange/index.php) (S2 Table). qRT-PCR assay was carried out according to the method described by Yang et al. [30]. There were four replicates for each qRT-PCR assay.

### Statistical analysis

Differences among treatments were analyzed with Student’s *t*-test with significance set to *P* < 0.05. The results were represented as the means ± SD for n = 3–8.

## Results

### Effects of P deficiency on the Plant DW and root/shoot ratio in *C*. *grandis*

**Fig 1:**
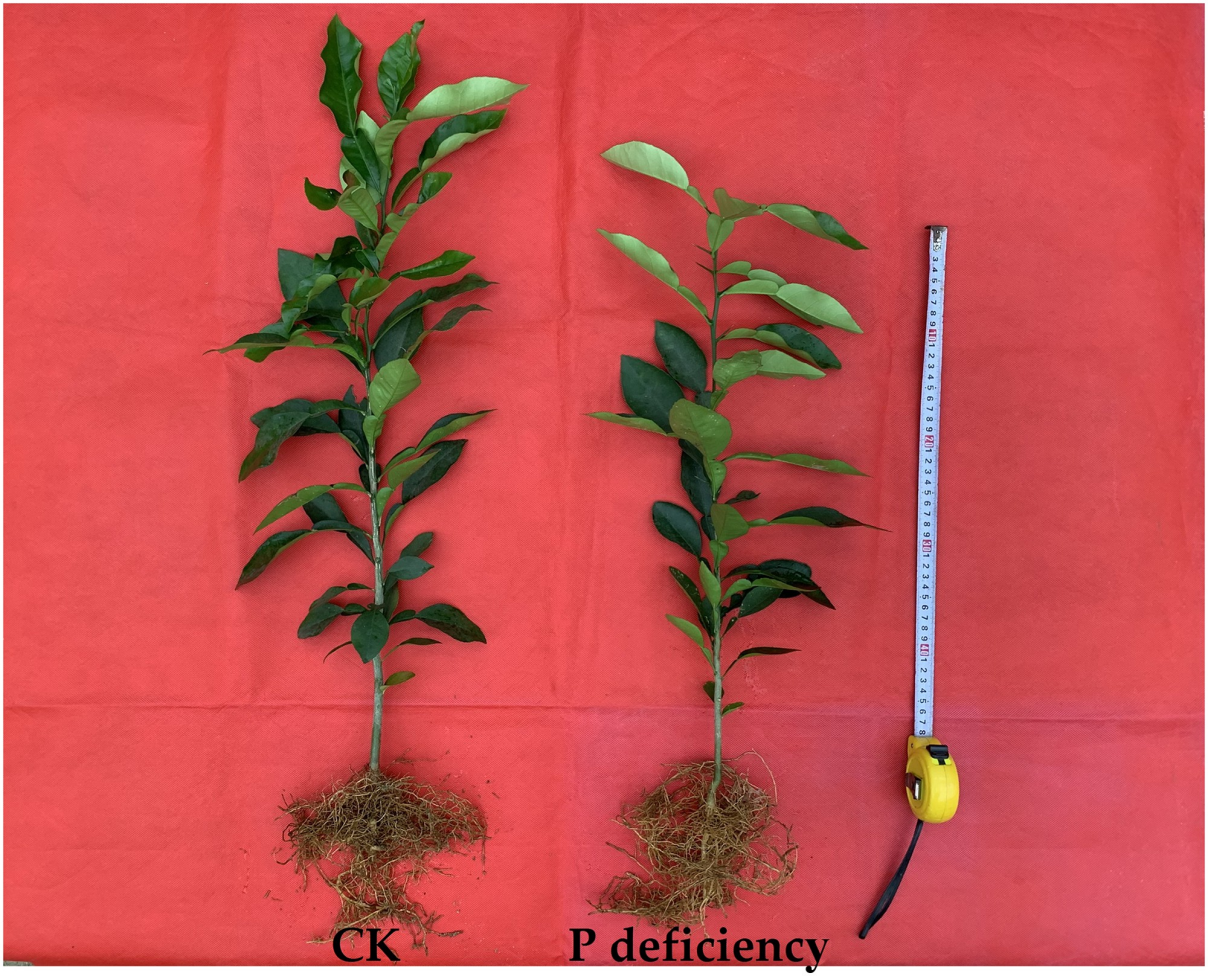
Effects of P deficiency on the growth of *C*. *grandis* seedlings.

**Fig 2:**
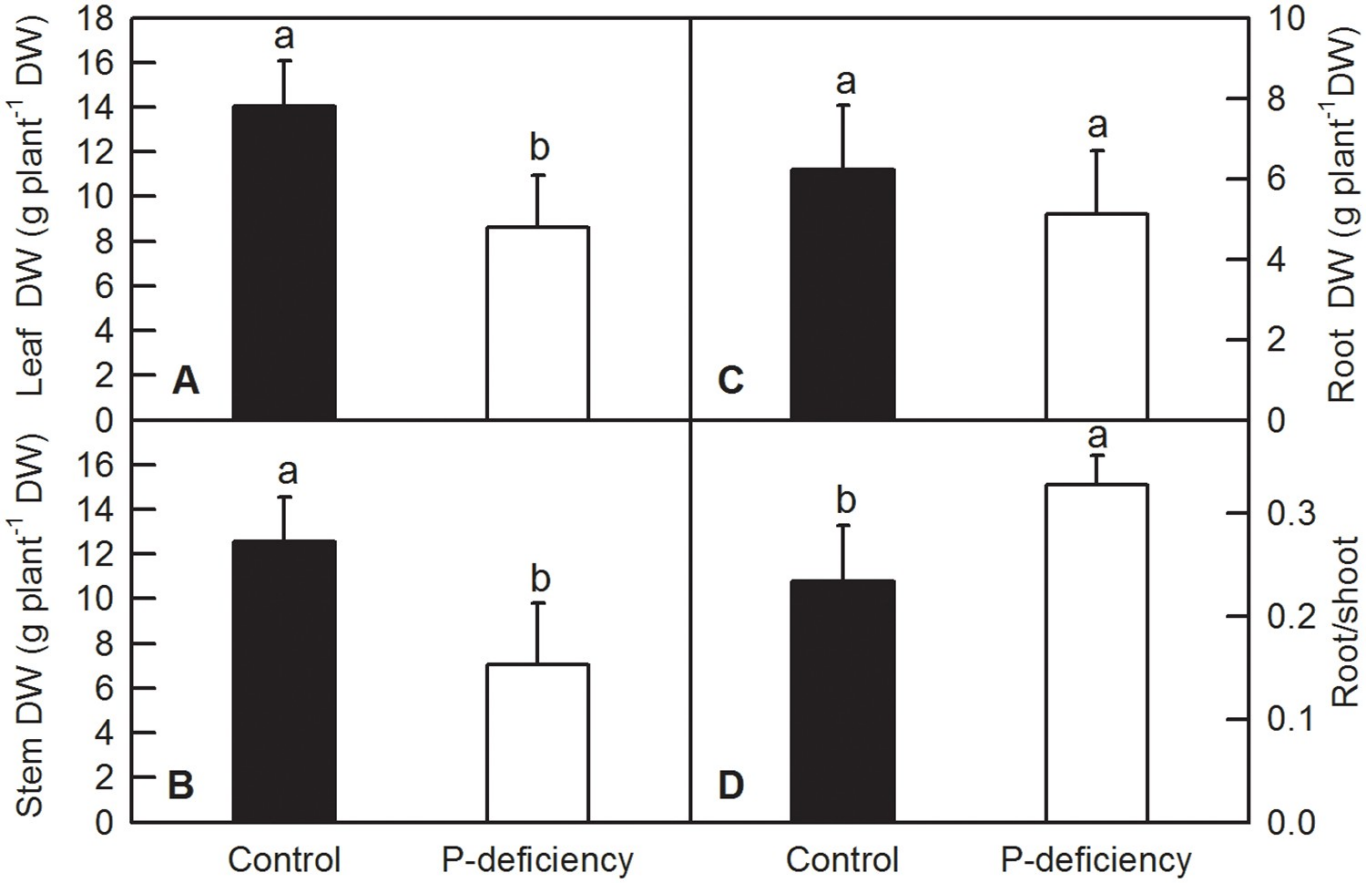
Effects of P deficiency on the biomass of *C*. *grandis*. Bars represent means ± SD (n = 8). Differences among the treatments were analyzed by *Student’s t*-test. Different letters above the bars indicate a significant difference at *P* < 0.05.

After 16 weeks of P deficiency treatment, roots, stems and leaves of *C*. *grandis* were harvested and oven dried. There was no obvious change in the appearance of *C*. *grandis* seedlings between control and P deficiency, except for shorter height of P-deficient seedlings than that of control seedlings (Fig 1). P deficiency significantly decreased the DW of leaves (Fig 2A) and stems (Fig 2B), while increasing the ratio of root to shoot DW (Fig 2D). P deficiency did not change the DW of roots (Fig 2C).

### Effects of P deficiency on the contents of mineral nutrients in leaves, stems and roots of *C*. *grandis*

**Fig 3:**
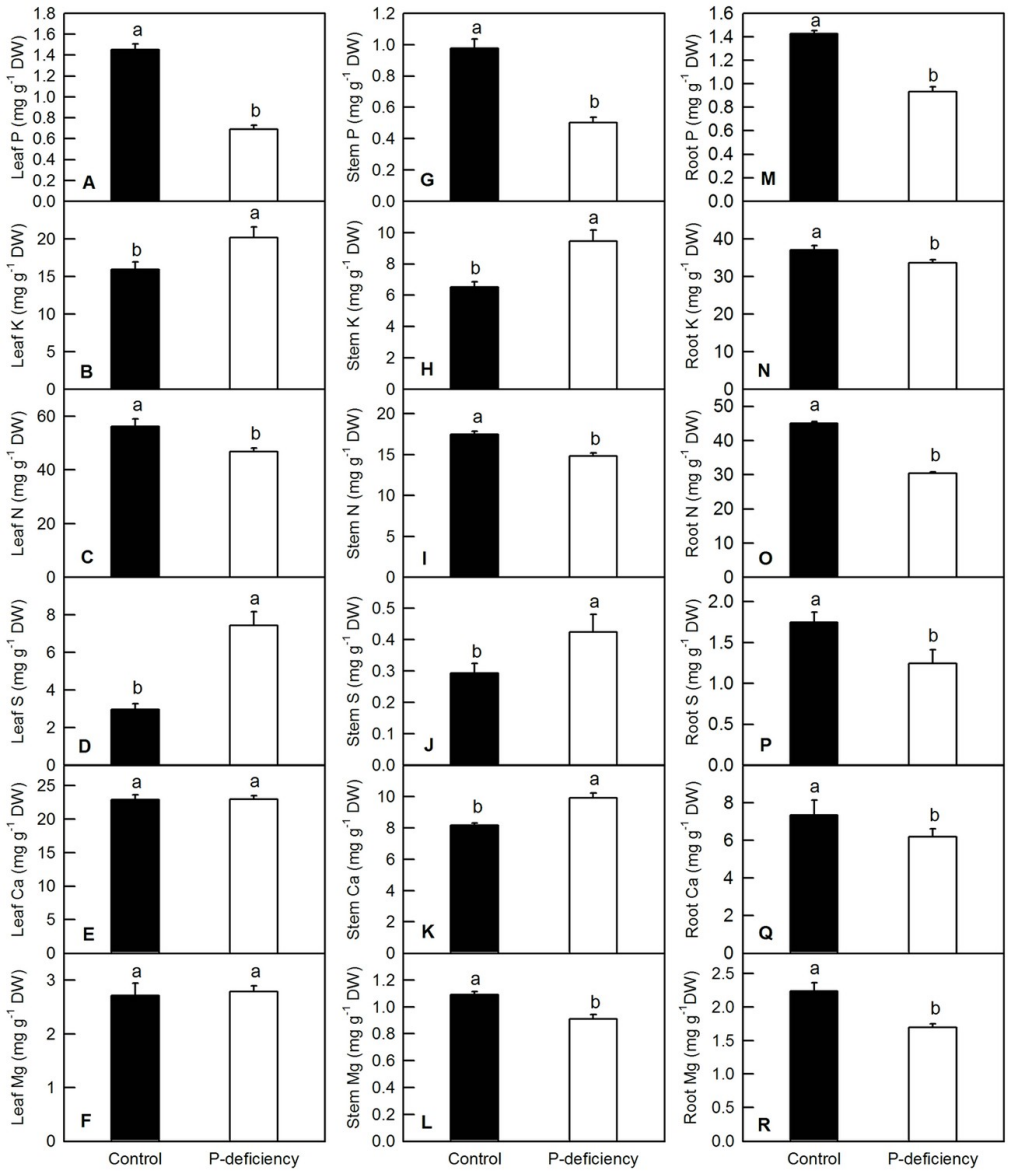
Effects of P deficiency on the contents of macronutrients in *C*. *grandis* leaves (A-F), stems (G-L) and roots (M-R). Bars represent means ± SD (n = 3). Differences among the treatments were analyzed by *Student’s t*-test. Different letters above the bars indicate a significant difference at *P* < 0.05.

**Fig 4:**
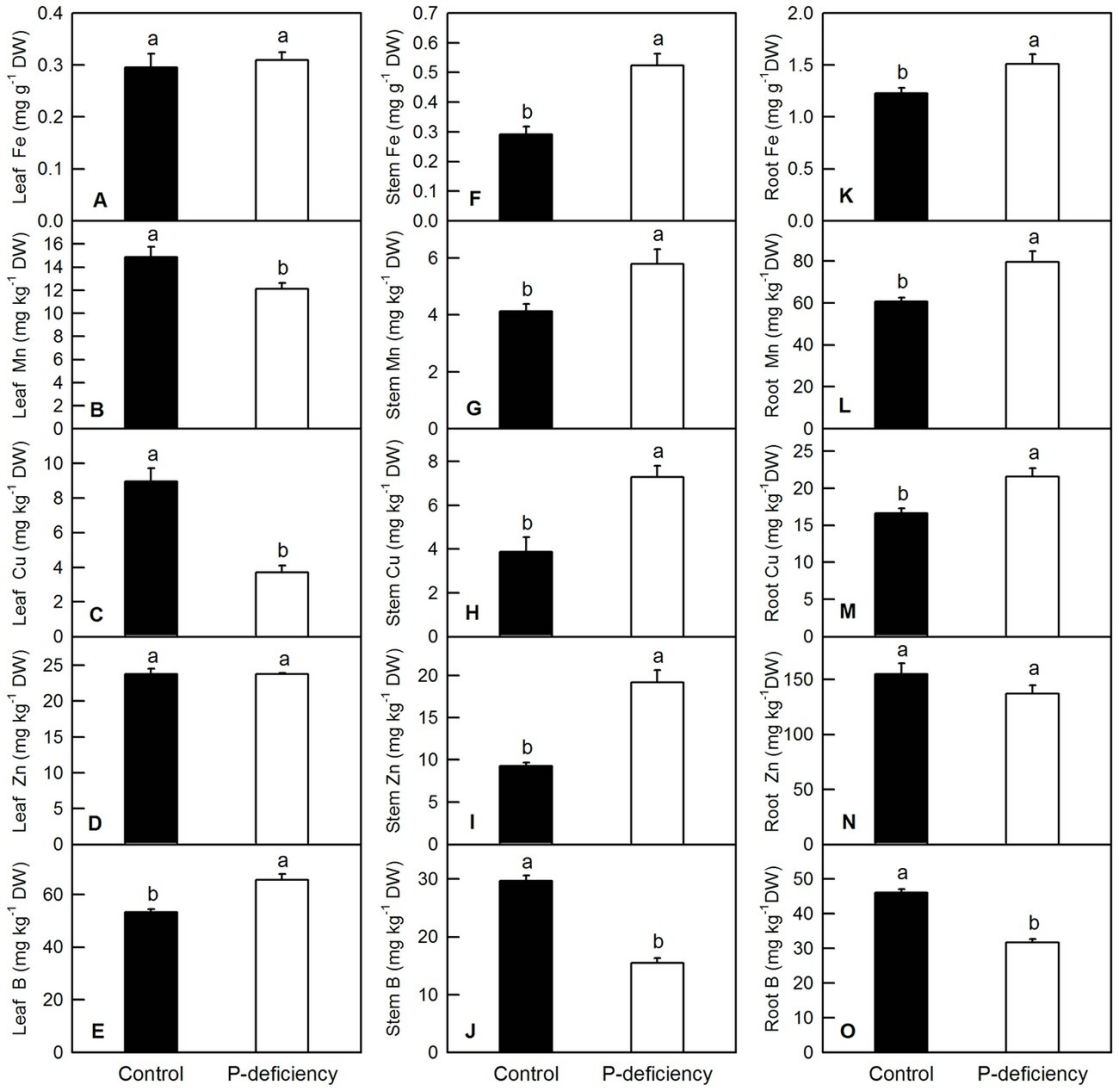
Effects of P deficiency on the contents of micronutrients in *C*. *grandis* leaves (A-E), stems (F-J) and roots (K-O). Bars represent means ± SD (n = 3). Differences among the treatments were analyzed by *Student’s t*-test. Different letters above the bars indicate a significant difference at *P* < 0.05.

For macronutrients, P deficiency in *C*. *grandis* decreased the contents of P and N in leaves and stems and the content of Mg in the stems (Fig 3A, 3C, 3G, 3I and 3L), whereas it increased the contents of K and S in leaves and stems and the content of Ca in the stems (Fig 3B, 3D, 3H, 3J and 3K). P deficiency did not change the contents of Ca and Mg in the leaves (Fig 3E and 3F). P deficiency significantly decreased the contents of P, K, N, S, Ca and Mg in the roots (Fig 3M–3R). For micronutrients, P deficiency did not change leaf Fe or leaf and root Zn (Fig 4A, 4D and 4N). P deficiency significantly increased leaf B (Fig 4E), stem Fe, Mn, Cu, and Zn (Fig 4F–4I), and root Fe, Mn, and Cu (Fig 4K–4M), whereas it decreased stem and root B in *C*. *grandis* (Fig 4J and 4O).

### Effects of P deficiency on gene expression levels of key channels related to K, B and S absorption and allocation in *C*. *grandis* roots

P deficiency did not affect the gene expression levels of *HKT2*, *AKT1* and *BOR1*, but significantly decreased that of *HKT1*, *SKOR*, *HSAT1* and *SAT1*.*3*. In contrast, P deficiency significantly increased the gene expression level of *NIP5*.*1* in *C*. *grandis* roots (S1 Fig).

### Effects of P deficiency on photosynthetic parameters and Chl content

**Fig 5:**
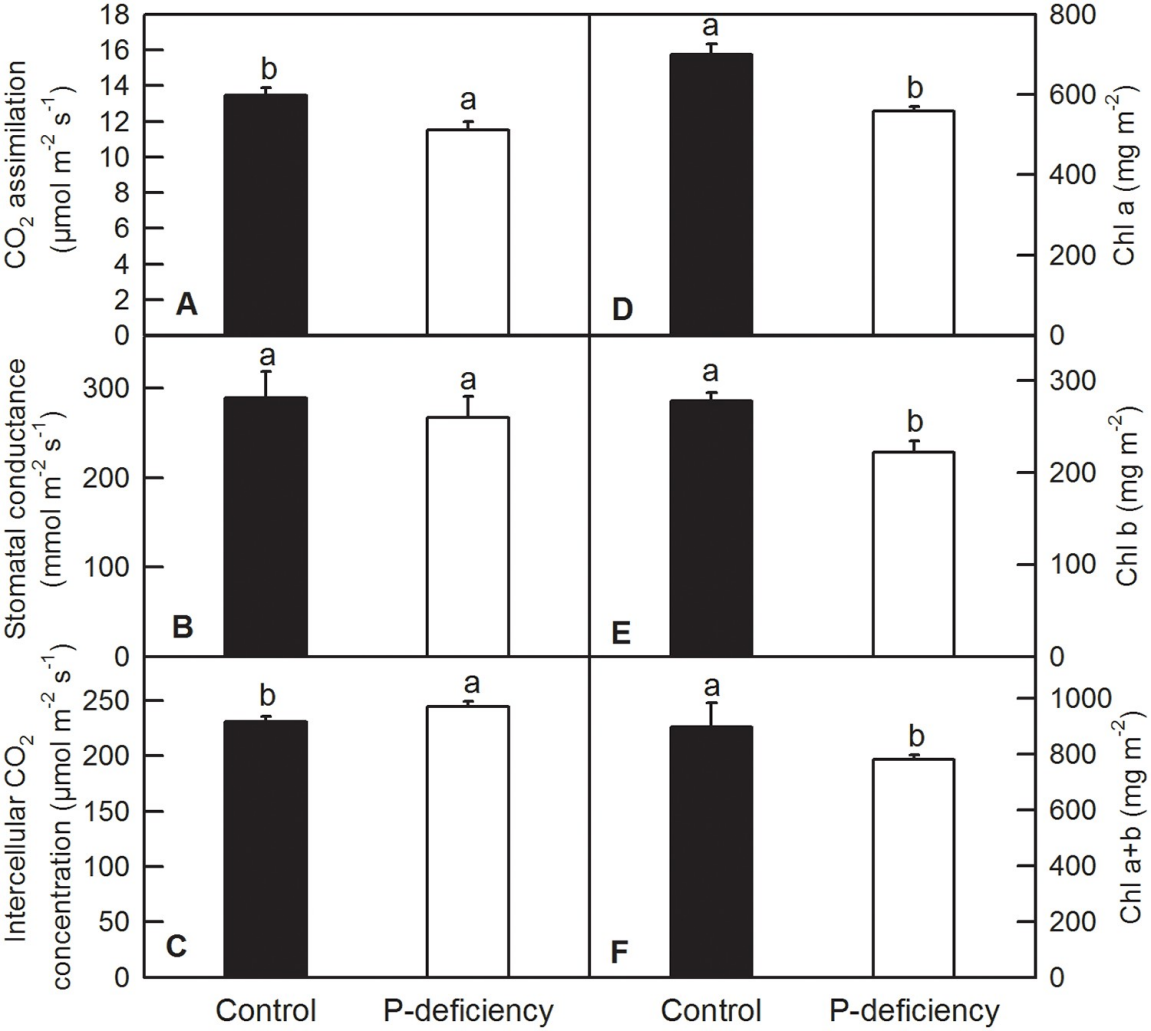
Effects of P deficiency on CO_2_ assimilation (A), stomatal conductance (B), and contents of intercellular CO_2_ (C), Chl *a* (D), Chl *b* (E) and Chl *a*+*b* (F). Bars represent means ± SD (n = 5 for A-C and 3 for D-F). Differences among the treatments were analyzed by *Student’s t*-test. Different letters above the bars indicate a significant difference at *P* < 0.05.

**Fig 6:**
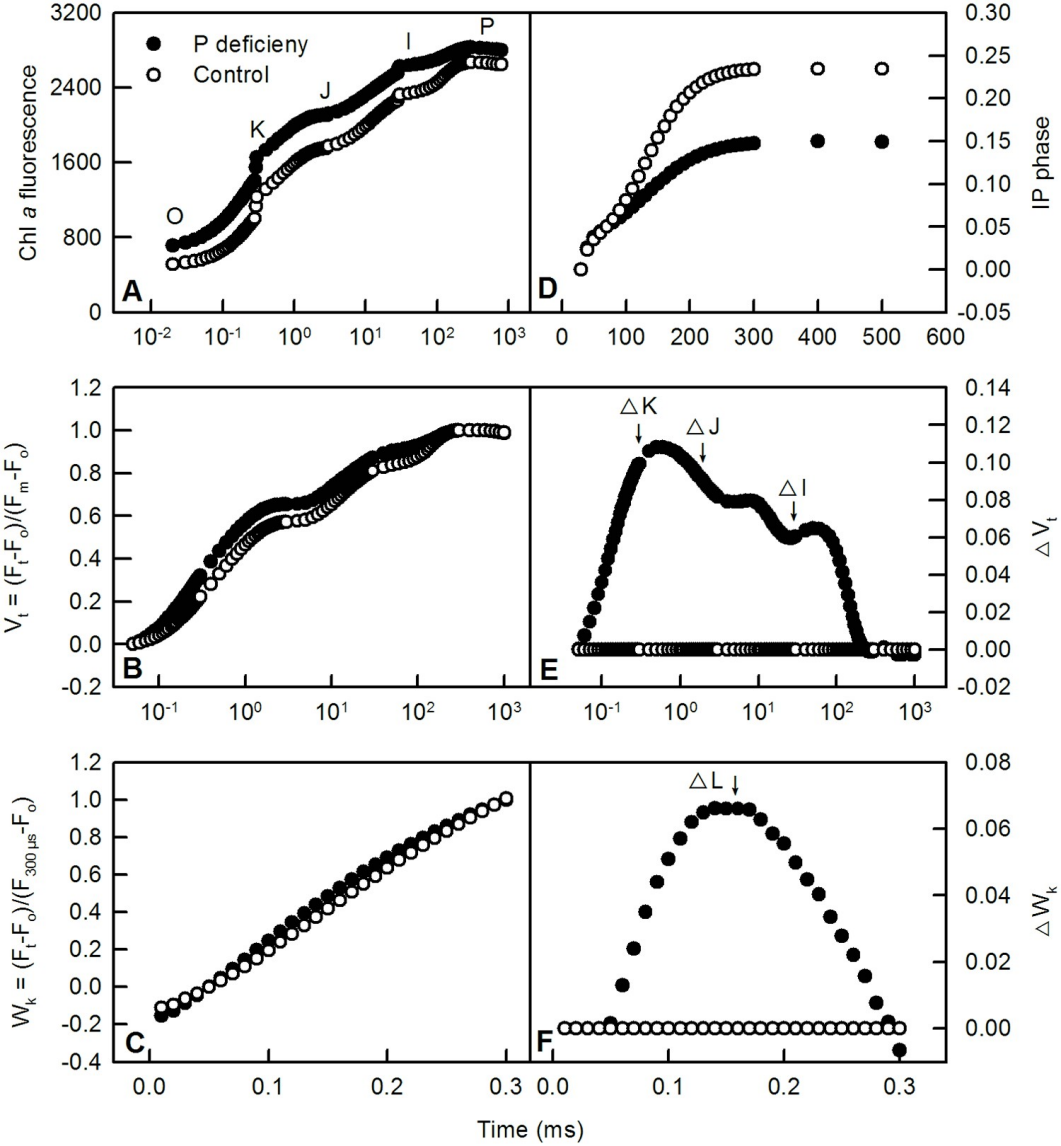
Effects of P deficiency on the average of Chl *a* fluorescence (OJIP) transients (A); relative variable fluorescence between F_o_ to F_m_, V_t_ = (F_t_-F_o_)/(F_m_-F_o_) (B) and F_o_ to F_300 μs_, W_k_ = (F_t_-F_o_)/(F_300μs_-F_o_) (C); IP phase from I-step to P-step, IP phase = (F_t_-F_o_)/(F_I_-F_o_)-1 = (F_t_-F_I_)/(F_I_-F_o_) (D), and the difference in V_t_ (△V_t_, E) and W_k_ (△W_k_, F) between P-deficient samples and control samples treated with 200 μM P in dark adapted leaves. Data are represented as the means of six replicates.

P deficiency decreased CO_2_ assimilation and increased the intercellular CO_2_ concentration in *C*. *grandis* leaves (Fig 5A and 5C), but did not change the stomatal conductance (Fig 5B). P deficiency decreased the contents of Chl *a*, Chl *b* and Chl *a*+*b* in leaves (Fig 5D–5F). Chl *a* fluorescence transient (OJIP test) measurement indicated that P-deficient and control leaves displayed a typical polyphasic fluorescence transient with clear O, K, J, I and P phases in both Chl *a* fluorescence transients and relative variable Chl *a* fluorescence transient (V_t_) (Fig 6A and 6B). P deficiency significantly decreased the IP phase [(F_t_-F_o_)/(F_I_-F_o_)-1 = (F_t_-F_I_)/(F_I_-F_o_)] in *C*. *grandis* leaves compared to that of control ones (Fig 6D). P-deficient leaves displayed a positive K-step, J-step, and I-step at approximately 300 μs, 2 ms and 30 ms, respectively, when the relative variable fluorescence (V_t_) was normalized to the control leaves. Furthermore, the △K was more pronounced than △J and △I (Fig 6E). The analysis of relative variable fluorescence between F_o_ and F_300μs_ (W_k_) and normalized W_k_ (△W_k_) compared with control leaves indicated that P-deficient leaves had a more pronounced L-band than the control leaves (Fig 6C and 6F). Analysis of selected parameters, yields and specific fluxes or activities showed that P deficiency significantly decreased the Area, RE_o_/RC, φP_o_, ψE_o_, φE_o_, φR_o_, RE_o_/CS_o_) and performance index (PI) for energy conservation from photons absorbed by PSII antenna to the reduction of Q_B_ (PI_abs_). P deficiency also increased F_o_, V_J_, V_I_, ABS/RC, ABS/CS_o_, DI_o_/RC, DI_o_/CS, TR_o_/RC, and TR_o_/CS (S2 Fig). However, P deficiency did not change F_m_, ET_o_/RC or ET_o_/CS in *C*. *grandis* leaves (S2 Fig).

### Effects of P deficiency on H_2_O_2_ production, anthocyanins and TBARS content in *C*. *grandis* leaves and/or roots

**Fig 7:**
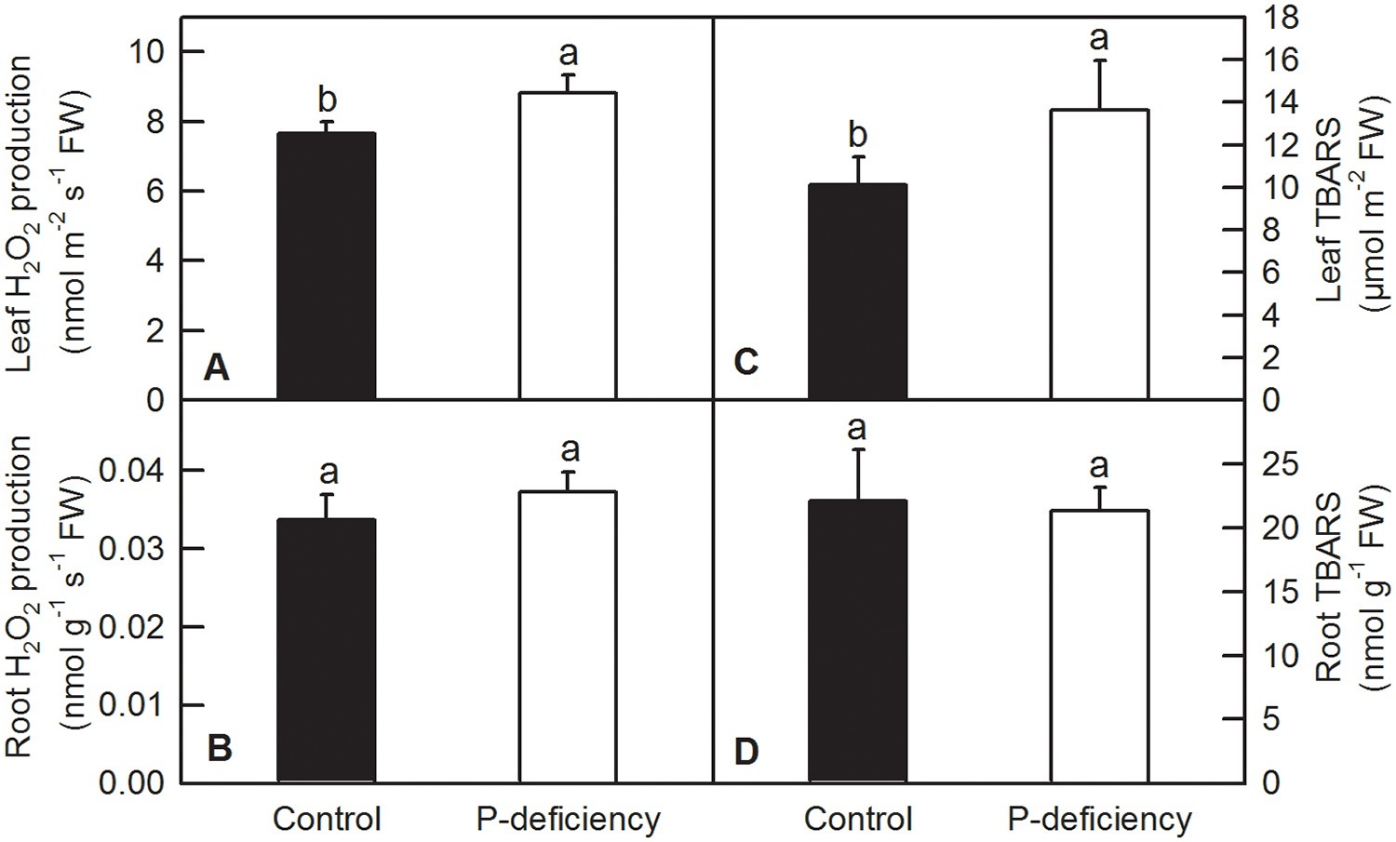
Effects of P deficiency on the production of H_2_O_2_ and the TBARS content in *C*. *grandis* leaves and roots. Each bar represents mean ± SD (n = 4). Differences among the treatments were analyzed by *Student’s t*-test. Different letters above the bars indicate a significant difference at *P* < 0.05.

P deficiency significantly increased H_2_O_2_ production and MDA content (TBARS) in *C*. *grandis* leaves (Fig 7A and 7C). No significant difference of anthocyanins content was observed between P deficient and control leaves of *C*. *grandis* (S3 Fig). In contrast, P deficiency treatment did not change H_2_O_2_ production and TBARS content in *C*. *grandis* roots (Fig 7B and 7D).

### Effects of P deficiency on antioxidant metabolite contents and the activities of antioxidant enzymes

**Fig 8:**
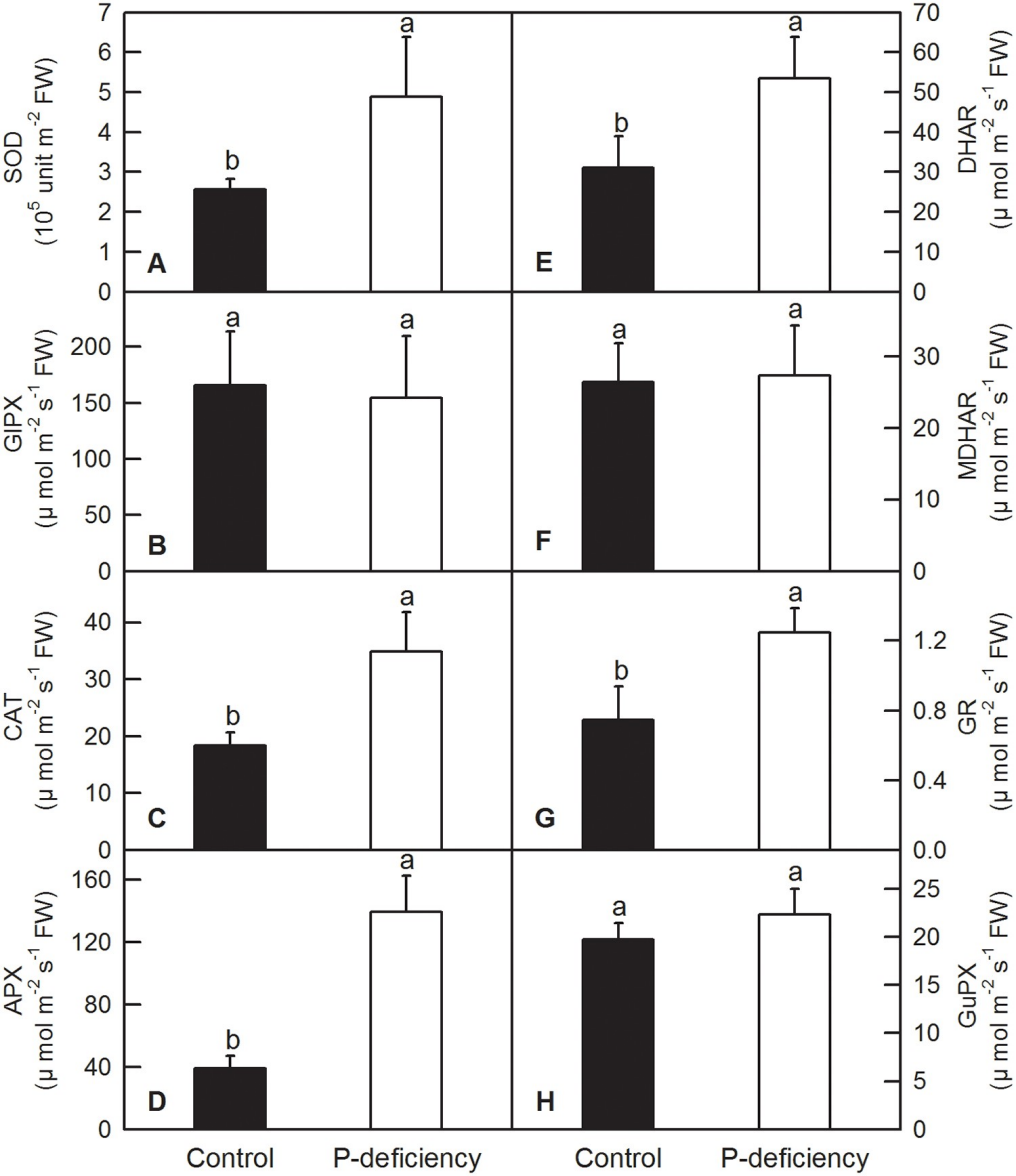
Effects of P deficiency on activities of antioxidant enzymes in *C*. *grandis* leaves. Each bar represents mean ± SD (n = 4). Differences among the treatments were analyzed by *Student’s t*-test. Different letters above the bars indicate a significant difference at *P* < 0.05.

**Fig 9:**
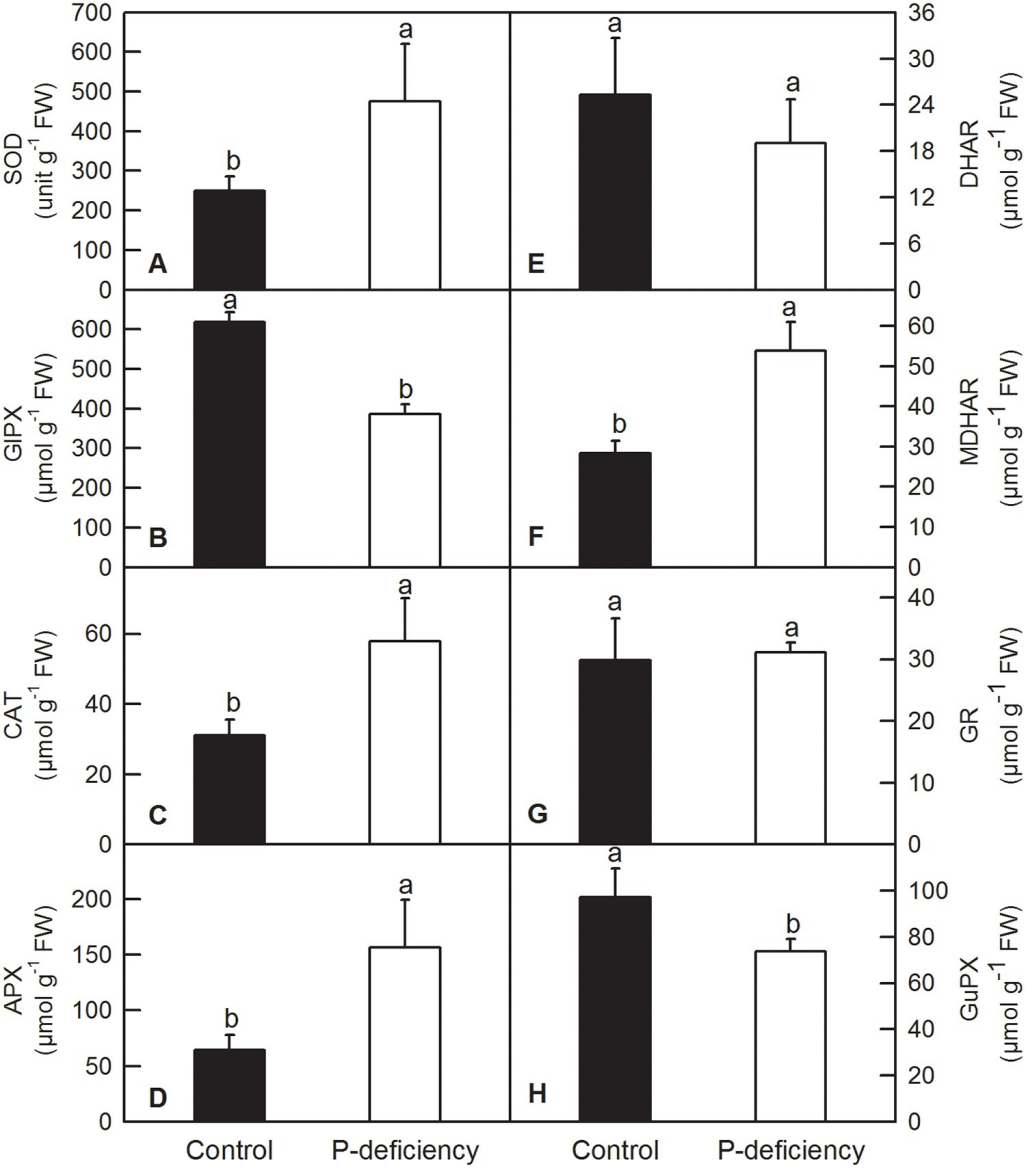
Effects of P deficiency on activities of antioxidant enzymes in *C*. *grandis* roots. Each bar represents mean ± SD (n = 4). Differences among the treatments were analyzed by *Student’s t*-test. Different letters above the bars indicate a significant difference at *P* < 0.05.

**Fig 10:**
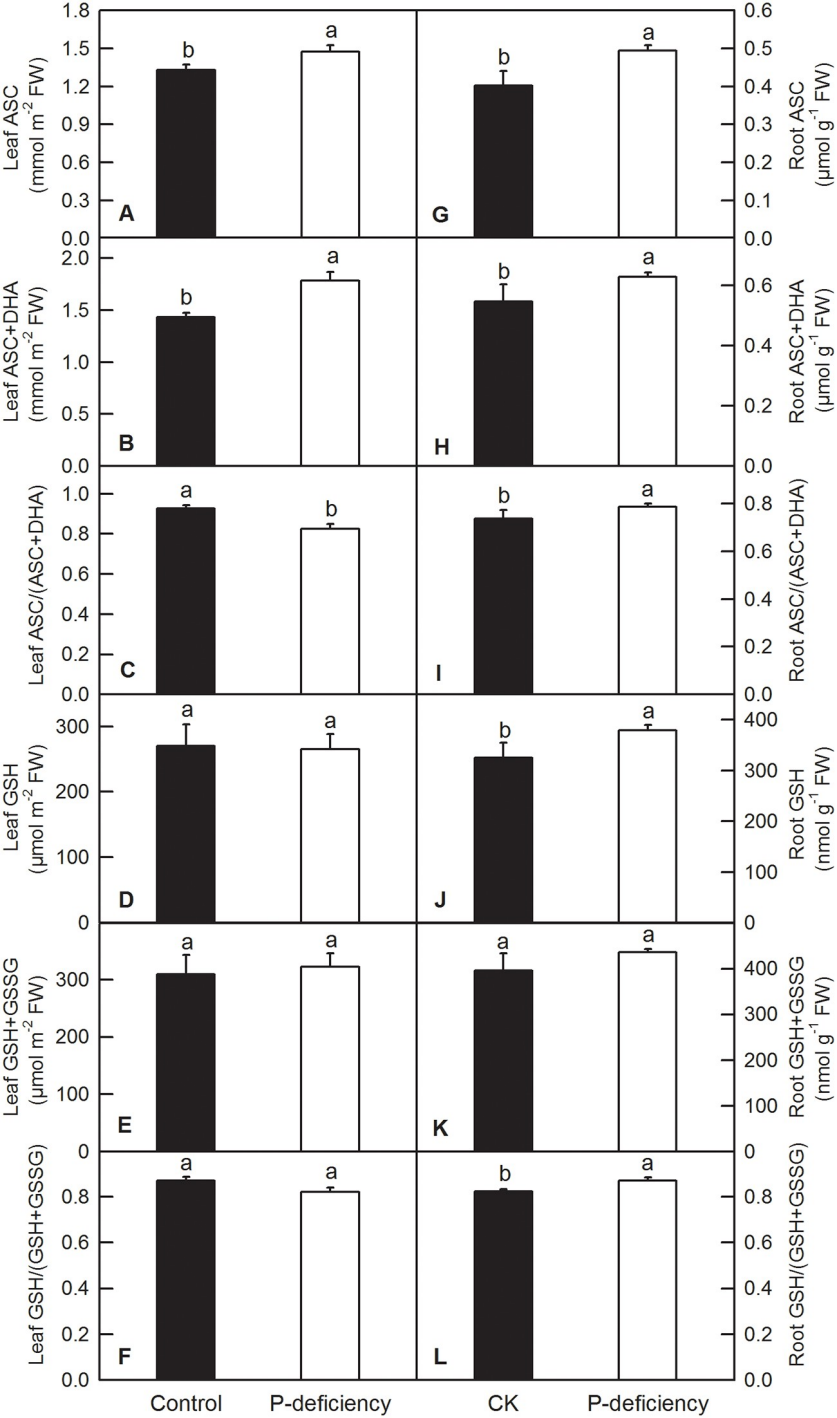
Effects of P deficiency on the contents of ASC, ASC+DHA, GSH, and GSH+GSSG, and on the values of ASC/(ASC+DHA) and GSH/(GSH+GSSG) in leaves (A-F) and roots (G-L) of *C*. *grandis*. Each bar represents mean ± SD (n = 4). Differences among the treatments were analyzed by *Student’s t*-test. Different letters above the bars indicate a significant difference at *P* < 0.05.

In plants, antioxidant enzymes and compounds play vital roles in the fluctuation and homeostasis of ROS. Our enzyme kinetics measurements showed that P deficiency significantly increased the activities of SOD, CAT, APX, DHAR and GR, whereas it did not change that of GlPX, MDHAR and GuPX in *C*. *grandis* leaves (Fig 8). P deficiency significantly increased the activities of SOD, CAT, APX and MDHAR and decreased the activities of GlPX and GuPX, whereas it did not change that of DHAR and GR in *C*. *grandis* roots (Fig 9). P deficiency increased the contents of leaf ASC, leaf ASC+DHA, root ASC, and root ASC+DHA and the ratio of root ASC to root ASC+DHA [root ASC/(ASC+DHA)], whereas it decreased the ratio of leaf ASC/(ASC+DHA) (Fig 10A–10C and 10G–10I). P deficiency did not change the contents of leaf GSH, leaf GSH+GSSG, the leaf GSH/(GSH+GSSG) ratio or root GSH+GSSG, but it increased the content of root GSH and the GSH/(GSH+GSSG) ratio (Fig 10D, 10E, 10J and 10K).

## Discussion

Low P has been shown to inhibit plant growth and decrease agricultural production in areas with low phosphorus fertilizer input. P deficiency led to a significant reduction in net photosynthesis rate and efficiency of the PSII reaction center in rice, sunflower, maize [7–9], sugar beet [10], soybean [11], tobacco [12], oat [5], sheepgrass [14], Tibetan wild barley [15] and tea [16], thereby significantly decreasing the DW of many crops [6]. As a result, the grain yield or production of crops is compromised by P deficiency [9]. The decreased DW of leaves and stems in *C*. *grandis* might be induced by decreased Chl content and CO_2_ assimilation, due to the impaired internal CO_2_ assimilation processes: the intercellular CO_2_ concentration was increased by P deficiency while the stomatal conductance was not changed (Figs 1, 2 and 5). Similarly, the decreased growth in shoots and increased root/shoot ratio induced by P deficiency were also observed in oat [5], tea [16], Chinese fir [28], bean [37], barley [26], *Stylosanthes* [52], strawberry [53], maize [54] and tomato [55].

P deficiency heterogeneously affected the nutrient contents in the leaves, stems and roots of *C*. *grandis*. For instance, P deficiency increased the contents of leaf K, S and B, whereas it decreased or did not affect the contents of other macro- and micronutrients in leaves of *C*. *grandis* (Figs 3A–3F and 4A–4E). This result indicated that P might have an antagonistic effect on leaf K, S and B. Similarly, the antagonistic effect of S and P fertilizer on the uptake and utilization of each other was conspicuous in straw as well as in grains of moong [56]. In tomato and other crops, B concentration in leaves was also reported to increase with decreasing P supply [57,58]. In *C*. *grandis* stems, P deficiency decreased the contents of P, N, Mg and B, but increased the contents of other macro- and micronutrients (Figs 3G–3L and 4F–4J). In roots, P deficiency decreased the contents of all the macronutrients and B, however, it increased the contents of Fe, Mn and Cu (Figs 3M–3R and 4K–4O). The decreased absorption of macronutrients and B might be induced by low sink demand and limited leaf expansion under P starvation as revealed by decreased DW of leaves and stems (Figs 1, 2, 3M–3R and 4K–4O) [12,27]. Interestingly, the increased contents of Fe, Mn and Cu in *C*. *grandis* roots (Fig 4K–4M) induced by P deficiency might be associated with the increased free cations of Fe, Mn and Cu, as phosphate can strongly chelate these ions. Similarly, depleting phosphate in the medium clearly resulted in an increase of Fe content in *Arabidopsis* seedlings [59]. Interestingly, the decreased root K and B could be consequence of the decreased expression levels of related channel genes, such as *HKT1*, *HKT2*, *AKT1* and *HSAT1*.*1* in *C*. *grandis* roots (S1 Fig). On the other side, the decreased root B could not be explained by the gene expression level of related channels, as P deficiency did not decreased the expression of *NIP5*.*1* and *BOR1* in *C*. *grandis* roots (S1 Fig) [60–62]. The increased shoot K, B and S can be partially due to the condensed effect as revealed by decreased shoot biomass (Figs 2–4). In brief, the altered nutrient contents arising from P deficiency might be due to the combination of altered root growth, rhizospheric ion reactions in the soil and lower sink demand of mineral nutrition for above-ground parts (Figs 1, 2A and 2B) [63].

The measurement of Chl *a* fluorescence transients (OJIP test) is a powerful non-intrusive method for monitoring changes in photochemical efficiency and photosynthetic electron transport [64–66]. Our Chl *a* fluorescence transient measurement showed that both control and P-deficient samples displayed a typical OJIP transient (Fig 6A and 6B). The decreased IP phase in P-deficient leaves might be due to the increased F_o_ rather than the decreased fluorescence from t = 30 ms to t = 500 ms (I step to P step)(Fig 6A and 6D; S2 Fig), which indicated that electron transport from the FeS clusters to ferredoxin and the reduction of plastocyanin (PC^+^) and P700^+^, and the cyclic electron transfer around PSI could be slowed down in P-deficient leaves [67–69]. Similar results were also observed in *C*. *grandis* under aluminum (Al) stress or manganese (Mn) excess, as well as tea under P deficiency and wheat under salinity stress and low nitrogen conditions [16,64,70,71].

The positive △K-band can be explained by an impaired electron flow from oxygen evolving complex (OEC) to the secondary electron donor (the tyrosine Z) of PSII, thus slowing down turnover of the reduction of Q_A_ [72], and/or changes in the architecture of PSII antenna that affect the energy migration properties within the photosynthetic unit (Fig 6E) [73]. According to the Grouping Concept [74] and JIP-test [75], a positive △L-band appeared at approximately 150 μs (Fig 6F), indicates that the PSII units were less grouped or energy transportation was hindered between independent PSII units in P-deficient leaves. The J-step and I-step of OJIP transients are correlated with the redox state of Q_A_ and the redox state of the plastoquinone pool. The positive △J- band and △I- band in the relative variable fluorescence, as well as increased V_J_ and V_I_, indicated that the electron flow from Q_A_ to Q_B_ and from plastoquinol (PQH_2_) to PSI were impaired (Fig 6E; S2 Fig), which resulted in a more reduced donor side and more oxidized acceptor side of PSII in P-deficient samples than in control ones [65,76,77]. The inhibition of electron flow from Q_A_ to Q_B_ (increased V_J_) of P-deficient samples was further complemented by the increased F_o_ and decreased PI_abs_, which might be due to the separation of LHC II from the PSII core complexes and the partly reversible inactivation of the reaction center of PSII revealed by decreased F_v_/F_m_ (S2 Fig) [78]. Interestingly, we found that P deficiency induced a more positive △K-band than △J- and △I- bands in *C*. *grandis* leaves (Fig 6E), demonstrating that P deficiency affected the donor side of PSII more than the acceptor side of PSII. This result was different from the changes of the variable fluorescence transients induced by Mn excess, Al toxicity and Mg deficiency in citrus plants [70,71,79].

Here, we showed that although P-deficient leaves captured more photon energy per reaction center (RC) and per cross-section (CS_o_), as indicated by the increased ABS/RC, ABS/CS_o_, TRo/RC and TR_o_/CS_o_, P deficiency decreased the electron transport efficiency from the donor side of PSII to the end acceptor side of PSI, revealed by decreased ψE_o_, φE_o_, φR_o_ and RE_o_/CS_o_, and dissipated more energy as heat or fluorescence (DI_o_/RC, DI_o_/CS_o_), leading to lowered CO_2_ assimilation in P-deficient *C*. *grandis* leaves (Fig 5; S2 Fig). An increase of ABS/RC, ABS/CS_o_, TR_o_/RC and TR_o_/CS_o_ has also been observed in other plants under drought stress, possibly due to inactivation of some PSII RCs or an increase in antenna size [64]. Similarly, the impaired electron transport from the donor side of PSII to the end acceptor side of PSI has also been found in wheat, rice and tea under P deficiency and in *C*. *grandis* or *C*. *sinensis* under Mn excess, Al toxicity and Mg deficiency [16,17,70,71,77].

Under P deficiency, more absorbed photon energy and fewer electrons used in the generation of reducing force inevitably lead to ROS production in *C*. *grandis* leaves. As expected, P deficiency significantly increased H_2_O_2_ production and TBARS content in *C*. *grandis* leaves (Fig 7A). As a strategic response, flexible regulation of antioxidant systems, such as ASC and GSH pools, and antioxidant enzymes, can be employed to counter adverse conditions, including P deficiency [16,30,80]. Here, we found that P deficiency significantly increased the contents of ASC and ASC+DHA but decreased the ASC/(ASC+DHA) ratio in *C*. *grandis* leaves (Fig 10A–10C). Despite the unchanged leaf GSH pool, increased ASC might facilitate the scavenging of H_2_O_2_ by APX, DHAR and GR via the ascorbate-glutathione cycle in *C*. *grandis* leaves (Fig 8D, 8E and 8G) [30]. Unfortunately, the elevated ASC pool and activities of antioxidant enzymes, such as SOD, CAT, APX, DHAR and GR, could not sufficiently eliminate the augmented ROS stress imposed by P deficiency in *C*. *grandis* leaves, as indicated by increased H_2_O_2_ production and TBARS content (Fig 7C). Consistent with our result, P deficiency significantly increased ASC content and APX activity in maize leaves; however, such responses could not eliminate the oxidative burst induced by P deficiency and eventually led to lipid peroxidation in leaf cells [9]. P-deficiency-induced increase in ROS burst and scavenging antioxidant systems has also been observed in rice [8,35,81], tomato [19], sheepgrass [14], *Arabidopsis* [36], *P*. *vulgaris* [37] and tea [38]. In contrast, P deficiency significantly increased the contents of ASC, ASC+DHA, GSH, and GSH+GSSG ASC/(ASC+DHA) and the enzyme activities of SOD, CAT, APX and MDHAR in *C*. *grandis* roots (Figs 9A, 9C, 9F, 9D and 10G–10K). The elevated levels of antioxidant compounds and enzymes conferred sufficient protection to *C*. *grandis* roots against oxidative stress induced by P deficiency, as indicated by the absence of an increase in H_2_O_2_ production and TBARS content in the root samples (Fig 7B and 7D).

## Conclusion

P deficiency significantly decreased the DW of leaves and stems and increased the root/shoot ratio, but did not affect the DW of roots in *C*. *grandis*. The decreased DW of leaves and stems in *C*. *grandis* might be caused by decreased Chl content and CO_2_ assimilation induced by P deficiency. P deficiency heterogeneously affected the nutrient contents in leaves, stems and roots, as well as increased root Fe, Mn and Cu. The Chl *a* fluorescence transient showed that P deficiency impaired electron transport from the donor side of PSII to the end acceptor side of PSI, but had a greater impact on the performance of the donor side of PSII than that of the acceptor side of PSII and PSI. Excess photon energy triggered oxidative stress in *C*. *grandis* leaves under P deficiency, whereas the elevated ASC pool and activities of SOD, CAT, APX, DHAR and GR could not provide sufficient protection to the leaves. In contrast, the elevated antioxidant compounds and enzymes, such as SOD, CAT, APX and MDHAR, protected *C*. *grandis* roots from oxidative stress induced by P deficiency, as indicated by no observed increase in H_2_O_2_ production and TBARS content in the root samples. Taking these results together, we conclude that P deficiency affects nutrient absorption and lowers photosynthesis performance, leading to ROS production, which might be the crucial cause of the inhibited growth of *C*. *grandis*.

## Supporting information

S1 Fig Effects of P deficiency on the gene expression levels of channels related to K, B and S absorption and allocation in *C*. *grandis* roots. (TIF)

S2 Fig The impact of P deficiency on the parameters and the behavior pattern from the donor side of PSII to the acceptor side of PSI. In the plot, each parameter was normalized to that of the control. Data are represented as the means of six replicates.
(TIF)

S3 Fig Anthocyanins contents in P deficient and control leaves of *C*. *grandis*. (TIF)

S1 Table Summary of parameters, formulae and their description using data extracted from Chl a fluorescence (OJIP) transient. (DOCX)

S2 Table Gene special primer pair used for qRT-PCR analysis. (XLSX)
