# Supplementary figures and images for: Effects of phosphorus deficiency on the absorption of mineral nutrients, photosynthetic system performance and antioxidant metabolism in Citrus grandis
Source: PLoS One. 2021 Feb 17;16(2):e0246944. doi: 10.1371/journal.pone.0246944 (PMC7888624; doi:10.1371/journal.pone.0246944)

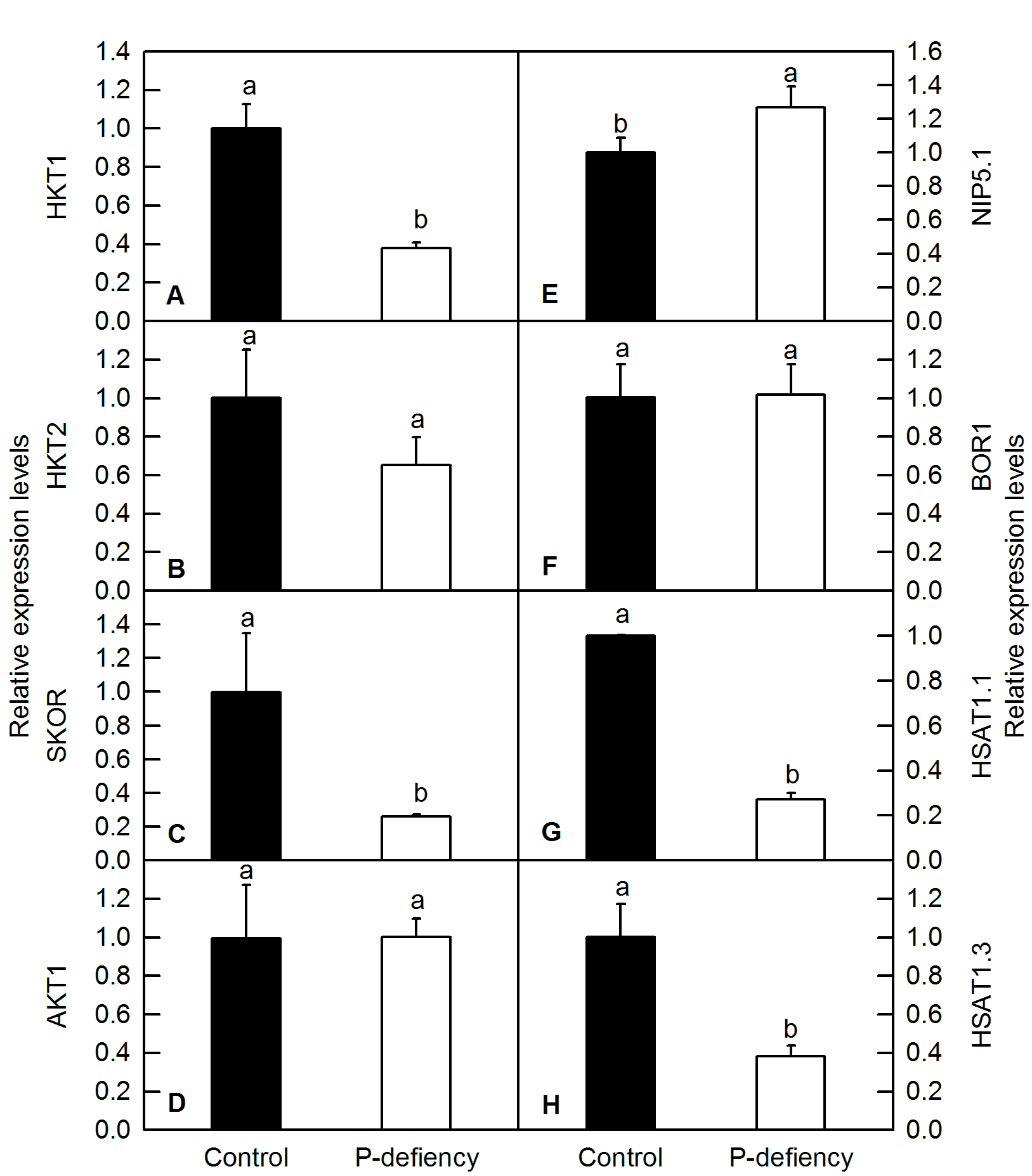

Supplement: S1 Fig — (TIF) [file pone.0246944.s001.Tif]

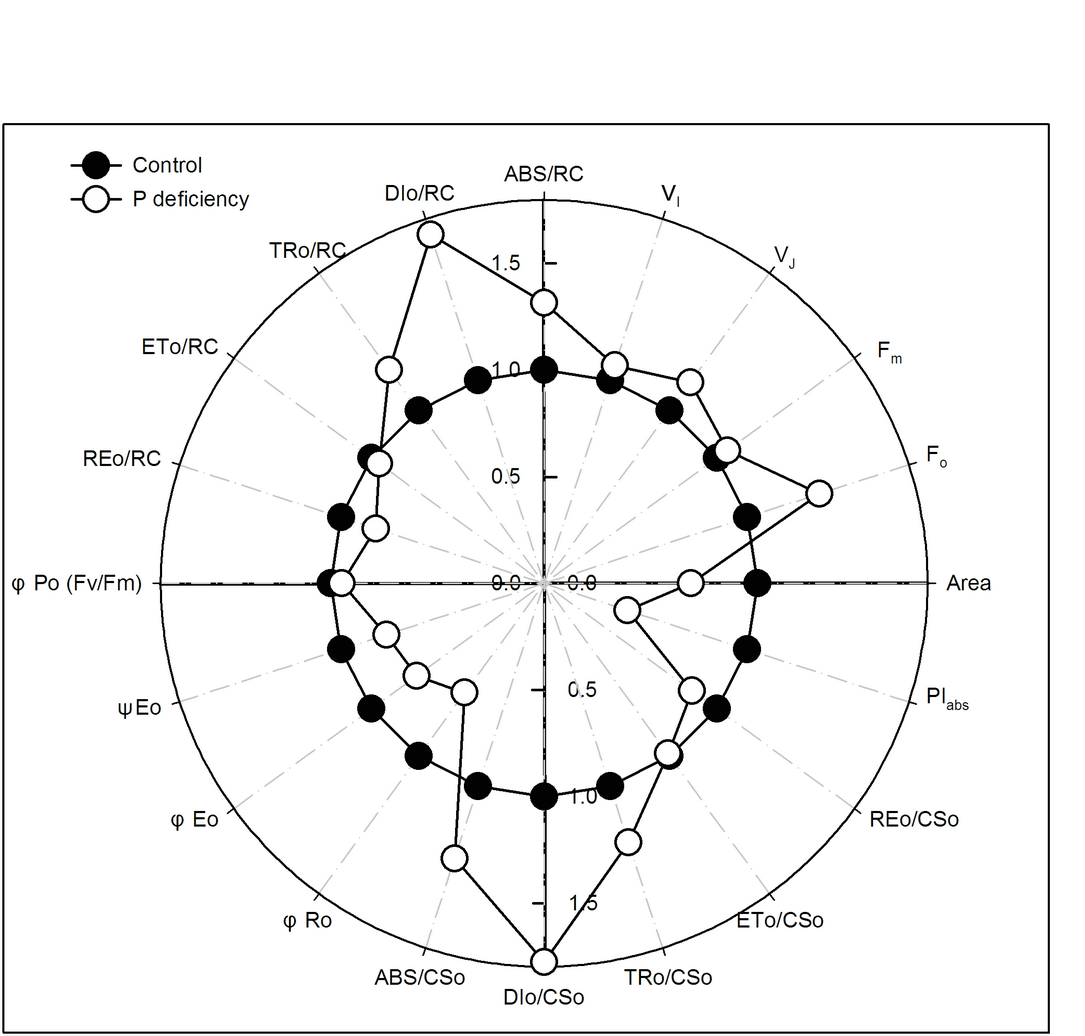

Supplement: S2 Fig — In the plot, each parameter was normalized to that of the control. Data are represented as the means of six replicates. (TIF) [file pone.0246944.s002.tif]

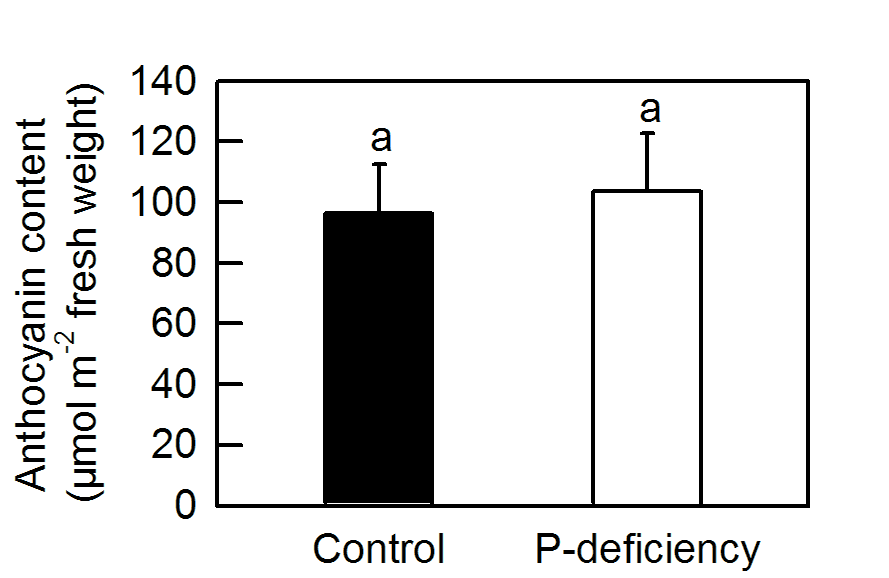

Supplement: S3 Fig — (TIF) [file pone.0246944.s003.Tif]
