# Supplementary material for: Effects of phosphorus deficiency on the absorption of mineral nutrients, photosynthetic system performance and antioxidant metabolism in Citrus grandis
Source: PLoS One. 2021 Feb 17;16(2):e0246944. doi: 10.1371/journal.pone.0246944 (PMC7888624; doi:10.1371/journal.pone.0246944)
Supplement: S1 Table — (DOCX) [file pone.0246944.s004.docx]

**S1 Table.** Summary of parameters, formulae and their description using data extracted from chlorophyll a fluorescence (OJIP) transient.

| **Fluorescence parameters** | **Description** |
| --- | --- |
| F_t_ | Fluorescence intensity at time t after onset of actinic illumination |
| F_50 μs_ | Minimum reliable recorded fluorescence at 50 μs with the PEA- or 20 μs with Handy-PEA-fluorimeter |
| F_300 μs_ | Fluorescence intensity at 100 and 300 μs, respectively |
| F_J_ and F_I_ | Fluorescence intensity at the J-step (2 ms) and the I-step (30 ms), respectively |
| F_P_ (= F_m_ ≅ F_500ms_) | Maximum recorded (= maximum possible) fluorescence at P-step |
| Area | Total complementary area between fluorescence induction curve and F = F_m_ |
| **Derived parameters** |  |
| **Selected OJIP parameters** |  |
| F_o_ ≅ F_50 μs_ or F_o_ ≅ F_20 μs_ | Minimum fluorescence, when all PSII RCs are open |
| F_m_ = F_P_ | Maximum fluorescence, when all PSII RCs are closed |
| V_J_ = (F_2 ms_ - F_o_)/(F_m_ - F_o_) | Relative variable fluorescence at the J-step (2 ms) |
| V_I_ = (F_30 ms_ - F_o_)/(F_m_ - F_o_) | Relative variable fluorescence at the I-step (30 ms) |
| M_o_ = 4 (F_300 μs_ - F_o_)/(F_m_ - F_o_) | Approximated initial slope (in ms^-1^) of the fluorescence transient V = f(t) |
| S_m_ = EC_o_/RC = Area/(Fm - F_o_) | Normalized total complementary area above the OJIP (reflecting multiple-turnover Q_A_ reduction events) or total electron carriers per RC |
| **Yields or flux ratios** |  |
| φP_o_ = TR_o_ABS = 1-(F_o_/F_m_) = F_v_/F_m_ | Maximum quantum yield of primary photochemistry at t = 0 |
| φE_o_ = ET_o_/ABS = (F_v_/F_m_) × (1 - V_J_) | Quantum yield for electron transport at t = 0 |
| ψE_o_ = ET_o_/TR_o_ = 1-V_J_ | Probability (at time 0) that a trapped exciton moves an electron into the electron transport chain beyond Q_A_^-^ |
| φD_o_ = DI_o_/ABS = 1-φP_o_ = F_o_/F_m_ | Quantum yield at t = 0 for energy dissipation |
| δR_o_ = RE_o_/ET_o_ = (1 - V_I_)/(1 - V_J_) | Efficiency with which an electron can move from the reduced intersystem electron acceptors to the PSI end electron acceptors |
| φR_o_ = RE_o_/ABS = φP_o_ × ψE_o_× δR_o_ | Quantum yield for the reduction of end acceptors of PSI per photon absorbed |
| **Specific fluxes or activities expressed per reaction center (RC)** | |
| ET_o_/RC = (M_o_/V_J_) × ψE_o_ = (M_o_/V_J_) × (1-V_J_) | Electron transport flux per RC at t = 0 |
| DI_o_/RC = (ABS/RC) - (TR_o_/RC) | Dissipated energy flux per RC at t = 0 |
| RE_o_/RC = (RE_o_/ET_o_) × (ET_o_/RC) | Reduction of end acceptors at PSI electron acceptor side per RC at t = 0 |
| ET_o_/CS_o_ = (ABS/CS_o_) × φE_o_ | Electron transport flux per CS at t = 0 |
| TRo/CS_o_ = (ABS/CS_o_) × φP_o_ | Trapped energy flux per CS at t = 0 |
| DI_o_/CS_o_ = (ABS/CS_o_) - (TR_o_/CS_o_) | Dissipated energy flux per CS at t = 0 |
| RE_o_/CS_o_ = (RE_o_/ET_o_) × (ET_o_/CS_o_) | Reduction of end acceptors at PSI electron acceptor side per CS at t = 0 |
| **Density of RCs** |  |
| RC/CS_o_ =φP_o_ × (ABS/CS_o_) × (V_J_/M_o_) | Amount of active PSII RCs per CS at t = 0 |
| **Performance index** | **Performance index (PI) on absorption basis** |
| PI_abs_ = (RC/ABS) × (φP_o_/(1 - φP_o_)) × (ψ_o_E/(1 - ψ_o_E)) | Performance index (PI) on absorption basis |
